# Discontinuing monoclonal antibodies targeting CGRP pathway after one-year treatment: an observational longitudinal cohort study

**DOI:** 10.1186/s10194-021-01363-y

**Published:** 2021-12-18

**Authors:** Fabrizio Vernieri, Nicoletta Brunelli, Roberta Messina, Carmelina Maria Costa, Bruno Colombo, Paola Torelli, Simone Quintana, Sabina Cevoli, Valentina Favoni, Florindo d’Onofrio, Gabriella Egeo, Renata Rao, Massimo Filippi, Piero Barbanti, Claudia Altamura

**Affiliations:** 1 Headache and Neurosonology Unit, Neurology, Campus Bio-Medico University Hospital, Via Alvaro del Portillo, 200, 00128 Rome, Italy; 2 Neurology, Neurorehabilitation and Neurophysiology Units, IRCCS Ospedale San Raffaele and University ‘Vita e Salute’, Milan, Italy; 3 Department of Medicine and Surgery, Parma and Neurology Unit, AOU di Parma, University of Parma, Parma, Italy; 4 IRCCS Istituto delle Scienze Neurologiche di Bologna, Bologna, Italy; 5 Neurology Unit, San Giuseppe Moscati Hospital, Avellino, Italy; 6 Headache and Pain Unit, IRCCS San Raffaele, Rome, Italy; 7 Neurology Unit, Department of Clinical and Experimental Sciences, University of Brescia, Brescia, Italy; 8 San Raffaele University, Rome, Italy

**Keywords:** Calcitonin gene-related peptide, Monoclonal antibodies, Migraine treatment, Real-world, Discontinuation

## Abstract

**Background:**

Monoclonal antibodies anti-calcitonin gene-related peptide (mAbs anti-CGRP) pathway are effective and safe on migraine prevention. However, some drug agencies limited these treatments to one year due to their high costs. This study aimed at evaluating the effect of discontinuing mAbs anti-CGRP on monthly migraine days (MMDs) and disability in high-frequency episodic (HFEM) and chronic migraine (CM) patients.

**Methods:**

This observational longitudinal cohort study was conducted at 10 Italian headache centres. Consecutive adult patients were followed-up for three months (F-UP1–3) after discontinuation of a one-year erenumab/galcanezumab treatment. The primary endpoint was the change in F-UP MMDs. Secondary endpoints included variation in pain intensity (Numerical Rating Scale, NRS), monthly acute medication intake (MAMI), and HIT-6 scores. We also assessed from F-UP1 to 3 the ≥50% response rate, relapse rate to CM, and recurrence of Medication Overuse (MO).

**Results:**

We enrolled 154 patients (72.1% female, 48.2 ± 11.1 years, 107 CM, 47 HFEM); 91 were treated with erenumab, 63 with galcanezumab. From F-UP1 to F-UP3, MMDs, MAMI, NRS, and HIT-6 progressively increased but were still lower at F-UP3 than baseline (Friedman’s analysis of rank, *p* < .001). In the F-UP1–3 visits, ≥50% response rate frequency did not differ significantly between CM and HFEM patients. However, the median reduction in response rate at F-UP3 was higher in HFEM (− 47.7% [25th, − 79.5; 75th,-17.0]) than in CM patients (− 25.5% [25th, − 47.1; 75th, − 3.3]; Mann-Whitney U test; *p* = .032). Of the 84 baseline CM patients who had reverted to episodic migraine, 28 (33.3%) relapsed to CM at F-UP1, 35 (41.7%) at F-UP2, 39 (46.4%) at F-UP3. Of the 64 baseline patients suffering of medication overuse headache ceasing MO, 15 (18.3%) relapsed to MO at F-UP1, 26 (31.6%) at F-UP2, and 30 (42.3%, 11 missing data) at F-UP3. Lower MMDs, MAMI, NRS, and HIT-6 and higher response rate in the last month of therapy characterized patients with ≥50% response rate at F-UP1 and F-UP3 (Mann-Whitney U test; consistently *p* < .01).

**Conclusion:**

Migraine frequency and disability gradually increased after mAbs anti-CGRP interruption. Most patients did not relapse to MO or CM despite the increase in MMDs. Our data suggest to reconsider mAbs anti-CGRP discontinuation.

## Background

Monoclonal antibodies targeting the calcitonin gene-related peptide (CGRP), i.e., mAbs anti-CGRP pathway, opened a new era in migraine prevention [1]. Randomized clinical trials (RCTs) demonstrated excellent effectiveness and safety of these treatments [2–7], and increasing real-world studies have confirmed this evidence in real life [8, 9]. International drug agencies have approved mAbs anti-CGRP to prevent episodic and chronic migraine in adults since 2018. However, because of the high costs of this therapeutic approach, national institutions set their own rules for their reimbursement to limit expenses. In Italy, anti-CGRP mAbs were initially administered on direct hospital dispensation in episodic migraine (EM) and chronic migraine (CM) patients, until the Italian Medicines Agency (AIFA) defined reimbursement criteria. Since July 2020, reimbursement criteria posed by AIFA to be respected also included a MIDAS score ≥ 11 when starting mAbs anti-CGRP and a mandatory improvement of that score of at least 50% after three months to continue. Moreover, Italian authorities currently allow treating both episodic and chronic patients for a maximum of one year, followed by a drug withdrawal of at least 3 months (reduced to one month on July 31st 2021). However, these reimbursement rules were set after Drug Agencies’ approval and before the real-world evidence.

Beyond limiting expenses and regulatory rules about discontinuing mAbs anti CGRP treatment, another important matter is whether those new drugs could modify the history of migraine disease.

Nowadays, we can learn from the real-life experience in the last two years prescribing CGRP targeted mAbs to refine treatment strategies. In particular, we can now provide evidence on the treatment discontinuation after one year. Is this approach correct? If so, how long should this discontinuation last? Should it be applied likewise to episodic and chronic patients? From a different point of view, discontinuing the mAbs anti-CGRP pathway is essential to understand if and to which extent these drugs can be disease-modifying. The study aimed to evaluate the effect of stopping anti-CGRP monoclonal antibodies on monthly migraine days and disability after one year of treatment in EM and CM patients.

## Methods

This observational longitudinal cohort study was conducted as part of two observational longitudinal multicentric studies on the real-life use of erenumab (the EARLY—ErenumAb in Real Life in ItalY—study) [8] and galcanezumab (the GARLIT—GalcanezumAb in Real Life in ITaly—study) [9], in 10 third level headache centres in Italy.

Consecutive patients diagnosed with episodic migraine with high-frequency (HFEM, i.e., 8–14 monthly migraine days, MMDs [10, 11]) or CM (1.3 ICHD-3 [12]), treated with mAbs anti-CGRP (erenumab or galcanezumab) from November 2019 to July 2021 according to clinical indication [13, 14], were considered. They were not previously involved in any randomized clinical mAbs anti-CGRP trial. Data collection of the EARLY and GARLIT studies is described elsewhere [8, 9]. The present paper considered the 12-month treatment and 3-month suspension interval regarding erenumab and galcanezumab patients’ data from the treatment phase on direct hospital dispensation. All enrolled patients discontinued the drugs for 90 days after one year of treatment, irrespective of the taken mAb.

In brief, monthly migraine days (MMDs), monthly acute medication intake (MAMI), monthly disability scale (i.e., Headache Impact Test- HIT-6, Italian version 1.1 [15]), and pain intensity (0–10 Numerical Rating Scale, NRS) of the monthly most painful attack were prospectively collected. All patients were educated on the headache diary use before enrolment in the EARLY and GARLIT studies. We included in the present study only patients who completed one-year mAbs anti-CGRP treatment followed by a 3-month suspension interval. During the 3-month suspension interval, patients attended clinical visits or were required to send monthly a copy of their headache diary (including MAMI and pain intensity) and HIT-6 scores by email.

The primary endpoint was to observe the change in MMDs in the three months following mAbs anti-CGRP discontinuation (F-UP 1–2-3) after one year of treatment compared with baseline and last month of mAbs treatment. Moreover, the changes in MMDs for each month in the follow-up period were also assessed as response rates (i.e., percentual reduction) compared to the pre-treatment period (baseline). We calculated the change in response rate as the absolute difference to the baseline from the last month of treatment to the 3rd month of follow-up. We also assessed the proportion of patients who could be still be classified as ≥50% responders at the follow-up evaluations. Secondary endpoints included changes in MAMI, in NRS, and HIT-6 score according to the same intervals. Besides, medication overuse (MO) was also considered in the follow-up period as the proportion of patients taking ≥15 NSAIDs or 10 triptans monthly. Among CM patients having episodic migraine at the end of the one-year treatment, we determined the proportion of patients presenting at 15 or more MMDs again at the follow-up evaluations. Finally, we observed the proportion of patients with at least 8 MMDs along evaluation times.

All patients provided written informed consent. The EARLY study received approval no.19/26 from the IRCCS San Raffaele Roma Institutional Review Board, while the GARLIT study was approved by the Campus Bio-Medico University Ethical Committee n.30/20. The other local Institutional Review Boards mutually recognized the approvals. The GARLIT study has been registered at the Italian Medicines Agency (Agenzia Italiana del Farmaco, AIFA) and at ClinicalTrials.govNCT04803513.

Anonymized data will be shared by request from any qualified investigator.

### Statistical analysis

Statistical analyses were performed with SPSS version 26.0 (SPSS Inc., Chicago, IL, USA).

This is a priori analysis. The sample size was considered in line with previous studies on the topic [16–20]. To achieve a power of 80% and a level of significance of 5% (two-sided), for detecting an effect size of 0.25 between paired variables, we calculated a sample size of at least 128 subjects. Interval variables were compared between groups with independent t-test (expressed as means with standard deviations [SD]) or Mann-Whitney tests (medians with 25th, 75th percentiles) according to the results of the Kolmogorov-Smirnov test for data distribution. Friedman’s analysis of rank was adopted to analyse the variable changes over time. We described proportions as percentages and categorical variables as frequencies. Contingency tables (Chi-square and two-tailed Fisher exact tests) were run to compare frequencies between groups. Statistical significance was set as two-tailed *p* < 0.05. Subjects with missing information regarding the main studied variables (MMDs, NRS, MAMI) were excluded. For the other variables, data availability has been declared.

## Results

Among patients enrolled in the EARLY and GARLIT studies, 154 patients (72.1% female, aged 48.2 ± 11.1 yrs., min-max 19–71 yrs) respected inclusion criteria. Of these, 107 patients were affected by CM and 47 by HFEM; 81 patients (69.2%) presented medication overuse headache (MOH) at baseline; 91 patients had been treated with erenumab and 63 with galcanezumab. The MMDs, pain intensity, and acute medication intake were available in all patients during the evaluation times, while HIT-6 scores were fully available in 71 patients (20 with HFEM and 51 with CM).

Table 1 summarizes demographical profiles and clinical variables evaluated at baseline, in the last month of treatment, and follow-up visits in CM and HFEM patients.

**Table 1:** Demographical profiles and clinical variables at evaluation times in HFEM and CM patients

	HFEM (***n*** = 47)	CM (***n*** = 107)
**Age** years, mean (SD)	48.9 (11.2)	49.4 (11.2)
**Sex** % (n females)	78.7 (37)	69.2 (74)
**BMI** kg/m^2^, median (25th,75th)	22.00 (20.43,23.63)	23.20 (22.00,25.70)
**Baseline MOH** % (n)	9.7 (10)	90.3 (93)
**Treatment** % (n Erenumab)	51.1 (24)	62.6 (67)
**MMDs** median (25th,75th)		
Baseline	11.0 (10.0,13.75)	20.0 (16.0,30.0)
last month	6.0 (4.0,9.0)	9.0 (5.7,14.3)
follow-up 1 month	9.0 (8.0,11.0)	14.5 (10.0,18.0)
follow-up 2 month	10.5 (9.2,11.7)	16.0 (10.7,20.0)
follow-up 3 month	10.5 (9.0,13.5)	15.0 (10.7,22.0)
**RR** median (25th,75th)		
last month	−63.6 (−84.7,-60.0)	−61.1 (-77.3, -40.0)
follow-up 1 month	−42.9 (−87.9,-42.5)	−46.6 (-69.6, -15.4)
follow-up 2 month	−28.6 (−71.5,–20.5)	−40.0 (-55.6, -10.0)
follow-up 3 month	−14.3 (−65.9,-10.3)	−30.0 (-55.0, -6.7)
last month - follow-up 3 difference	−47.7 (−79.5,-17.0)	−25.5 (−47.1,-3.3)
**MPI** median (25th,75th)		
Baseline	12.0 (10.5,14.5)	21.0 (15.0,32.0)
last month	4.0 (2.0,7.0)	7.0 (4.0,13.0)
follow-up 1 month	6.0 (2.5,11.0)	8.0 (5.0,15.0)
follow-up 2 month	9.0 (5.5,12.5)	12.0 (8.0,18.0)
follow-up 3 month	10.0 (6.0,13.5)	15.0 (9.0,22.0)
**NRS** median (25th,75th)		
Baseline	7.5 (7.0,8.0)	7.0 (7.0,8.0)
last month	6.0 (4.0,6.2)	5.0 (4.0,7.0)
follow-up 1 month	6.0 (4.7,7.0)	6.0 (4.0,7.0)
follow-up 2 month	6.0 (4.7,7.0)	6.0 (5.0,7.0)
follow-up 3 month	7.0 (5.0,7.0)	7.0 (5.0,8.0)
**Hit-6** median (25th,75th)[n]		
Baseline	66.5 (64.7,70.0) [33]	67.0 (64.0,72.0) [83]
last month	51.0 (41.5,60.0) [24]	55.0 (50.0,62.0) [57]
follow-up 1 month	56.0 (31.5,62.3) [20]	60.0 (53.0,66.0) [51]
follow-up 2 month	61.5 (51.5,65.0) [22]	62.0 (53.5,66.5) [51]
follow-up 3 month	60.5 (48.3,65.3) [37]	63.0 (51.0,68.0) [75]

After the three-month discontinuation (Table 1), F-UP3 MMDs, acute medication intake, pain intensity, and HIT-6 were still lower than baseline (Friedman’s analysis of rank; consistently, *p* < .001).

**Fig. 1:**
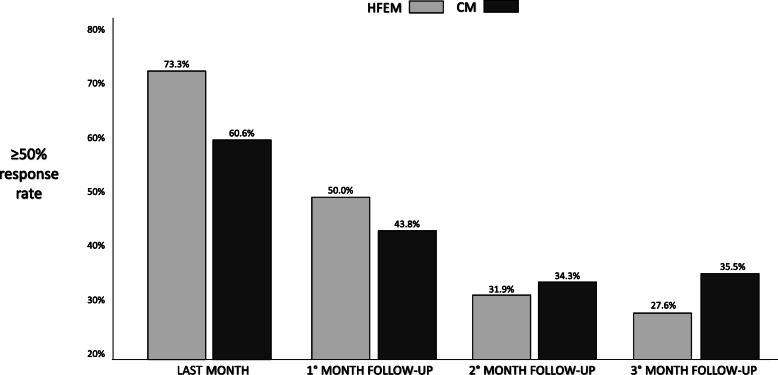
The frequencies of patients with ≥50% response rate at the last month of therapy with mAbs anti-CGRP pathway and at the three-months of discontinuation in CM and HFEM

Figure 1 shows the frequency of patients with ≥50% response rate in CM and HFEM groups in the last month of therapy and at F-UP1–3 visits. No difference was observed in ≥50% response rate frequency between CM and HFEM patients along the 3 evaluation times (χ2; consistently *p* > .100).

**Fig. 2:**
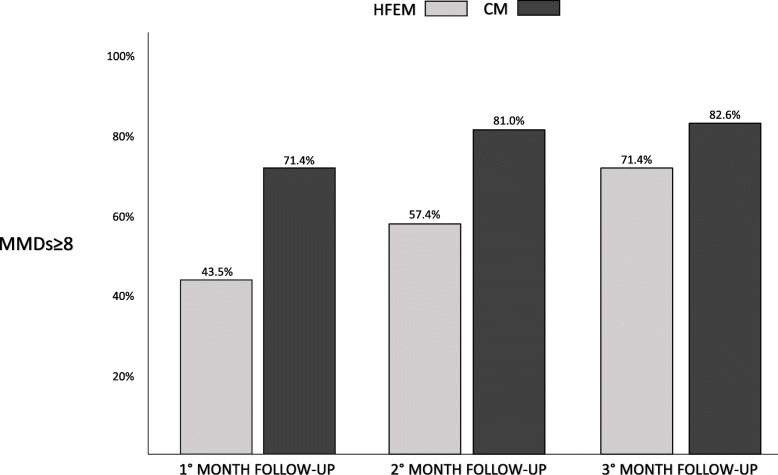
The frequency of patients having at least 8 MMDs in the three months of mAbs anti-CGRP pathway discontinuation in CM and HFEM patients

Figure 2 shows the frequency of patients having at least 8 MMDs in CM and HFEM groups. No significant difference was observed between CM and HFEM patients, although the comparison for the first month of follow-up was nearly significant (Mann-Whitney U test; F-UP1: *p* = .054; F-UP2: *p* = .692; F-UP3: *p* = .144).

**Fig. 3:**
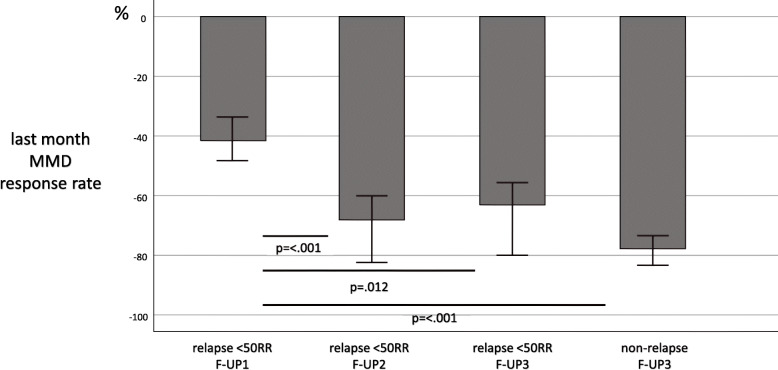
The median value (95% CI bars) of MMDs percentual reduction in the last month of therapy in patients relapsing to < 50% response rate at F-UP1, F-UP2, F-UP3, and patients still responding at F-UP3 (Mann-Whitney U test)

**Fig. 4:**
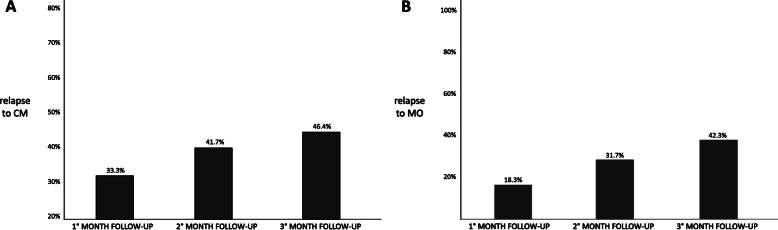
The frequency of patients relapsing to CM (A) and MO (B) MMDs in the three months of mAbs anti-CGRP pathway discontinuation

Table 2 compares the assessed variables according to long-lasting ≥50% response rate after one (F-UP1) and three (F-UP3) months of discontinuation. As evidenced, patients experiencing a long-lasting F-UP1 and F-UP3 response had lower MMDs, acute medication intake, pain intensity, and HIT-6 and higher response rate in the last month of treatment compared with non long-lasting responders (Mann-Whitney U test, consistently *p* < .001). On the contrary, baseline characteristics, including the type of monoclonal antibody, did not differentiate the responder and non-responder groups at F-UP1 and F-UP3. Figure 3 shows the median value (95% CI bars) of MMDs percentual reduction (response rate) in the last month of therapy in patients relapsing to < 50% response rate response at F-UP1, F-UP2, F-UP3, and patients still responding at F-UP3 (Mann-Whitney U test). At the end of the one-year treatment, 84 (78.5%) baseline CM patients presented episodic migraine, 64 (79.0%) patients had discontinued MO. Figure 4 displays the rate of relapse to CM (A) and MO (B) in these groups of patients.

**Table 2:** Demographical profiles and clinical variables at evaluation times in patients with ≥50%RR at F-UP1 and F-UP3 (i.e. long-lasting responders) compared with non-lasting responders

	F-UP1 ≥50%RR (***n*** = 70)	F-UP1 no 50%RR (***n*** = 84)	p	FUP-3 ≥50%RR (***n*** = 51)	F-UP3 no %RR (***n*** = 103)	p
**Age** years, mean (SD)	49.1 (10.8)	49.7 (11.4)	.746	47.7 (11.6)	49.8 (11.0)	.298
**Sex** % (n females)	71.4 (50)	75.3 (61)	1.000	76.4 (39)	69.9 (72)	.313
**BMI** kg/m^2^, median (25th,75th)	22.80 (20.85, 25.92)	22.60 (21.00, 24.50)	.752	23.40 (21.30, 26.40)	22.07 (20.93, 24.00)	.110
**CM % (n)**	67.1 (47)	71.4 (60)	.595	74.5 (38)	66.9 (69)	.438
**Baseline MO** % (n)	67.1 (47)	66.7 (56)	1.000	76.4 (39)	62.1 (64)	.131
**Treatment** % (n Erenumab)	59.4(41)	61.0 (50)	.489	50.9 (26)	63.1 (65)	.195
**MMDs** median (25th,75th)						
Baseline	16.0 (12.0,25.0)	15.5 (12.8,20.0)	.928	15.0 (12.0,25.0)	15.0 (11.0,22.5)	.545
Last month	4.0 (2.0,6.5)	9.0 (5.0,14.0)	**<.001**	3.5 (2.0,7.0)	6.0 (4.0,12.5)	**<.001**
**Last month RR** median (25th,75th)	−77.3 (−85.4,-60.0)	−41.9 (−63.6,-21.1)	**<.001**	−80.0 (− 86.7,-62.5)	−56.7 (−72.2,-31.7)	**<.001**
**MPI** median (25th,75th)						
Baseline	18.0 (12.0,30.0)	18.0 (13.0,29.3)	.912	18.0 (13.0,30.0)	15.0 (12.0,30.0)	.594
Last month	4.0 (2.0,7.0)	9.0 (5.0,13.0)	**<.001**	3.0 (1.0,6.0)	8.0 (4.0,12.0)	**<.001**
**NRS** median (25th,75th)						
Baseline	7.0 (6.0,8.0)	7.0 (7.0,8.0)	.226	7.0 (6.0,8.0)	7.0 (7.0,8.0)	.887
Last month	5.0 (3.0,6.0)	6.0 (5.0,7.0)	**<.001**	4.0 (3.0,6.0)	6.0 (5.0,7.0)	**<.001**
**HIT-6** median (25th,75th)						
Baseline	69.0 (64.0,72.5)	66.0 (65.0,69.0)	.052	69.0 (64.0,73.0)	66.0 (64.0,70.0)	.075
Last month	52.0 (39.0,59.0)	58.0 (53.8,62.3)	**.001**	44.0 (38.0,54.0)	57.0 (53.3,62.0)	**<.001**

## Discussion

In the present study, we aimed at evaluating the effect of discontinuation of mAbs anti-CGRP (erenumab and galcanezumab) after 12 months of therapy on monthly migraine days, symptomatic drugs intake, pain severity, and disability in high-frequency episodic migraine and chronic patients. While several RCTs and real-life studies have provided consistent data on the efficacy of this therapy, several questions have yet to be solved regarding its long-term effect on disease history and the rapidity of efficacy vanishing.

Our study demonstrated that MMDs and the other considered parameters increased gradually and consistently during the 3 months after discontinuation, both in HFEM and CM patients. Accordingly, 50% response rate decreased during 3-month discontinuation in both groups. The wearing-off seemed larger in HFEM patients, which experienced a reduction in ≥50% response rate from 73.3% at the 12th month of therapy to 27.6% after three months of discontinuation (Fig. 1). In the CM group, it gradually diminished from 60.6% to 35.5% at the end of the third month of suspension. On the other side, 71,4% of CM patients presented at least 8 MMDs already at F-UP1, making them again clinically eligible for the treatment, while the same percentage was observed in HFEM patients only at F-UP3.

Randomized clinical trials have poorly addressed the issue of mAbs discontinuation. A gradual reduction of effect on MMDs change after stopping galcanezumab was found in a review of data from the 3 randomized placebo-controlled trials [21]. However, the 4 months of discontinuation followed different treatment periods: a 6-month double-blind period in EM patients and a 3-month double-blind plus a 9-month open-label extension period in CM patients.

The effect of discontinuing mAbs anti-CGRP was evaluated in different studies considering small samples of patients [17–20], mostly treated only with erenumab. In each of those studies, MMDs and acute medications intake gradually increased after stopping treatment compared with the last month of mAbs anti-CGRP therapy, in the early phase (4 weeks) [18] and after three months of evaluation [17, 19, 20].

In the study by Gantenbein et al. [20], MMDs were still reduced in the third month after the last dose only in a small proportion (25%) of patients. The authors stated that the therapeutic effect of anti-CGRP antibodies outlasting their pharmacological effect is very limited.

More recently, Raffaelli et al. [16] confirmed that the cessation of mAbs targeting the CGRP pathway determines an increase in migraine frequency and acute medication intake in patients treated with mAbs targeting CGRP receptor (erenumab) or ligand (fremanezumab and galcanezumab). Moreover, the authors observed a faster MMDs increase in patients treated with the former than with the latter, probably related to the shorter elimination half-life of erenumab. However, this difference was only temporary as migraine frequency was back to baseline the fourth month after discontinuing all the three mAbs.

In our sample, more than one-third of CM subjects still benefitted from halving of attacks after three months of discontinuation. This represents an important issue as these patients had been very disabled, with at least three failed preventives, and felt relief from their migraine for the first time. No effectiveness or disability parameter per se can fully show the benefit in such resistant patients after taking a treatment capable for the first time to improve their quality of life even if mildly [22]. One could anticipate that these patients assume to encounter a worsening of their headache rapidly following the pre-imposed stop of three months (nocebo effect). In this line, we could also have expected a rebound increase in MMDs derived at least in part from upregulation of the CGRP receptors during the treatment period [23].

Beyond analyzing the mere increase of MMDs during the 3-month suspension and the reduction of the percentage of patients with ≥50% response rate, the data collected in our 154 patients have other aspects worth highlighting. About 80% of the 84 baseline CM patients reverted to EM at the end of the 12-month mAbs anti-CGRP treatment. Less than half of these patients relapsed to CM up % at F-UP-3. Moreover, of the patients who had ceased MO (around 80%) after one year of treatment, only 1/3 returned to overuse acute medication after the 3rd month of discontinuation (Fig. 4). One interpretation of these long-term beneficial effects is that weaning the brain from migraine pain by acting in the periphery may have central effects resetting the system to a lower pain load. In support of this hypothesis, in a mouse model of chronic migraine, repeated nitroglycerin (NTG) administration significantly increased the number of CGRP-R and pituitary adenylate-cyclase activating polypeptide (PACAP)-R neurons in trigeminal ganglion [24]. In this line, a functional magnetic resonance study observed a decreased activation of different structures of the pain network in patients with a positive response to erenumab administration [25]. To note all studies addressing mAbs antiCGRP withdrawal reported a load of MMDs after three months of interruption lower than baseline. One could also speculate that the persisting beneficial effect can derive from the reversal of migraine-driven vicious circles affecting different aspects of a migraineur’s life, e.g. lifestyle and psychosocial situations. Once migraine attacks have decreased, patients can enjoy more likely a healthier lifestyle, being less impacted by psychosocial stress [26]. Interestingly, a longer-lasting benefit (50%response rate at F-UP visits) was not related to clinical baseline characteristics (migraine frequency and disability) or the type of anti-CGRP or CGRP receptor antibody, but to the extent of the beneficial effect obtained in the one-year treatment (last month MMDs and response rate, Table 2 and Fig. 3), suggesting that the more effective is the therapy, the more incisive and longer is the impact on migraine.

However, to understand if a disease-modifying effect exists, it would be necessary to observe the clinical course of patients withdrawing the therapy for longer than five half-lives. Unfortunately, this cannot be currently achieved in a real-life observational study.

Our and other evidence from real-life settings may help answer some questions about mAbs anti-CGRP discontinuation and its effect. Our data demonstrated that their therapeutic effect does not outlast after discontinuation in most patients even after a long-term, i.e., one-year, treatment. We know that the half-life of mAbs varies from 26 to 32 days [27], with their plasma concentration reducing exponentially in the following weeks till reaching one-eights of their initial concentration after 3 months of discontinuation [28]. Our and other real-world evidence (RWE) translate the pharmacokinetic properties of these drugs in clinical terms as the effectiveness in terms of MMDs reduction and percentages of patients with ≥50% response rate in most HFEM and CM patients already decreases after one month and gradually and consistently throughout the 3-month discontinuation.

Accordingly, these should not be considered disease-modifying treatments, at least after a one-year administration, as far as we know, but as therapies that have to be continued to maintain their effectiveness, at least in most disabled patients. Nevertheless, some benefits persisted in our and other RWE, especially in CM and MO patients, even considering that these are often subjects with a long disease history and many preventives’ failures.

Our data seem not to support the appropriateness of treatment discontinuation after 12 months and a fixed interruption of 3 months in all patients undergoing mAbs anti-CGRP treatment. Most patients of our sample rapidly returned eligible to mAbs anti-CGRP. More practically, since many CM patients with a very high frequency presented at least 10 MMDs and severe disability (60 points at HIT-6 score) at the end of the first month of interruption, one could wonder if it is appropriate to undergo discontinuation after one year of treatment.

Chronic migraine is a very disabling condition, often comorbid with other disorders [29], deeply affecting patients’ emotional and cognitive spheres and different life aspects, including their occupation and career progression, partnership, family planning, and parenting, with dramatic social disadvantage [30]. So that, it is inadequate to impose an interruption, even if of only one month, of the therapy that most of these patients believe is the first effective if we do not have enough data in support. Also, since we observed a relapse to CM and MO at F-UP3 in less than half of patients treated for one year, we may wonder if a more extended treatment regimen would exert a more significant persistent effect after discontinuation.

In HFEM patients, our findings show contrasting approaches. If, on the one hand, the wearing-off effect seemed more prominent than in CM (Fig. 1), on the other hand, because of their lower baseline MMDs, HFEM subjects become eligible again to mAb anti-CGRP in a lower proportion than CM at the F-UP times (Fig. 2). Nevertheless, at F-UP2, more than half of HFEM patients were again eligible. So that, in HFEM patients, discontinuation could be appropriate to verify when attacks’ frequency again reaches the threshold of high-frequency migraine, making necessary to undergo a preventive treatment,

Long-term studies have demonstrated the safety of erenumab in patients treated up to 5 years [31]. Thus medicine regulation agencies based their decisions on discontinuation mainly on cost-saving. Even if RCTs remain the most appropriate way of evaluating the efficacy of therapeutic interventions, real-life studies have become increasingly important in the scientific world in recent years. Although their numerous limitations, they have the advantage of better representing the population we usually have to deal with in our everyday clinical practice. Different RWE about mAbs has provided several helpful data to improve migraine patients’ management with these new treatments [8, 9, 32]. We have to consider real-life experiences to improve our clinical practice, having the possibility to choose the best management beyond the a priori imposed rules.

Future studies are necessary to explore if any clinical characteristic influence the outcome of mAb anti-CGRP discontinuation to individuate subgroups of patients that can stand therapy withdrawal with lower consequences.

## Data Availability

Anonymized data will be shared by request from any qualified investigator.

## Abbreviations

AIFA: Agenzia Italiana del Farmaco
CI: confidence intervals
CGRP: calcitonin gene-related peptide
CM: chronic migraine
EM: episodic migraine
F-UP: follow-up
HFEM: high frequency episodic migraine
HIT-6: Headache Impact Test 6 items
MMDs: Monthly Migraine Days
mAb: monoclonal antibody
MO: medication overuse
MAMI: Monthly Acute Medication Intake
NRS: Numerical Rating Scale
MIDAS: Migraine Disability Assessment
RCT: randomized controlled trial
RWE: real-world evidence
